# Maternal adverse childhood experiences and epigenetic aging in pediatric populations: a scoping review

**DOI:** 10.3389/frcha.2026.1913117

**Published:** 2026-10-05

**Authors:** Debjani Chanda, Munawar Hussain Soomro, Jaeden Cole, Gillian England-Mason, Deborah Dewey

**Affiliations:** 1 Bachelor of Health Sciences Program, Cumming School of Medicine, University of Calgary, Calgary, Alberta, Canada; 2 Department of Pediatrics, Cumming School of Medicine, University of Calgary, Calgary, Alberta, Canada; 3 Owerko Centre, Alberta Children’s Hospital Research Institute, University of Calgary, Calgary, Alberta, Canada; 4 Medical Sciences Graduate Program, University of Calgary, Calgary, Alberta, Canada; 5 Department of Community Health Sciences, Cumming School of Medicine, University of Calgary, Calgary, Alberta, Canada; 6 Hotchkiss Brain Institute, University of Calgary, Calgary, Alberta, Canada

**Keywords:** epigenetic aging, epigenetic clocks, maternal ACEs, pediatric populations, scoping review

## Abstract

**Introduction:**

Maternal exposure to adverse childhood experiences (ACEs) may influence aging biomarkers in pediatric populations, as estimated using epigenetic clocks or other measures of aging, such as telomere length. This scoping review aimed to identify and map the available evidence on the association between maternal ACEs and biomarkers of epigenetic aging in offspring from birth to 18 years of age.

**Methods:**

A systematic search of MEDLINE, Embase, APA PsycINFO, CINAHL, Scopus, and ProQuest Dissertations and Theses Global was conducted on 26 May 2025 and updated on 28 April 2026. The inclusion criteria were epigenetic clock studies that included pediatric populations (0–18 years of age) born to mothers who self-reported ACEs during gestation or within 1 year after childbirth using a standard questionnaire. DNA methylation analysis was conducted using biospecimens, and offspring aging biomarkers were examined. Three reviewers independently screened 1,441 articles, of which 38 underwent full-text review.

**Results:**

Four studies met our inclusion criteria. The geographic scope of the studies was limited; three studies were conducted in the United States and one in the United Kingdom. Sample sizes ranged from 80 to 883 maternal-child dyads. The age at the time of biological sample collection ranged from birth to 8 years. Measures of epigenetic aging were examined across diverse tissue types, including blood, saliva, and umbilical vein endothelial cells. Some evidence has been found for an association between maternal ACEs and offspring epigenetic aging; however, the findings were often limited to specific conditions or ACE subtypes. Total maternal ACE scores were not significantly associated with newborn gestational epigenetic age acceleration or child symptoms. However, more specific associations emerged for maternal experiences of parental divorce, sexual abuse, and neglect. In addition, sex-specific associations were also noted. Racial disparities were also evident with maternal childhood abuse linked to increased epigenetic aging in Black but not White maternal-child dyads. Some associations emerged only when considering moderators such as maternal restless sleep during pregnancy.

**Discussion:**

Maternal ACEs may influence offspring epigenetic aging; however, these associations are complex, context-dependent, and supported by a limited body of evidence. Longitudinal studies with greater demographic diversity and methodological consistency, alongside mechanistic investigations of the biological pathways linking maternal adversity and offspring epigenetic aging, are needed.

## 1 Introduction

Early life, particularly the prenatal and early postnatal periods, represents critical windows of development during which environmental exposures can have profound and lasting effects on biological systems. Exposure to adversity during these developmental periods may lead to persistent alterations in body structure, metabolic processes, neurodevelopment, and physiological function (1, 2). Early life adversity has been associated with a broad range of adverse health outcomes across the life course, including obesity, type 2 diabetes, cardiovascular disease, certain cancers, osteoporosis, neurodegenerative diseases, reproductive dysfunction, asthma, autoimmune conditions, and mental health disorders, such as depression and schizophrenia (3). Increasing evidence suggests that some of these long-term associations may arise through biological processes that influence the timing and trajectory of development and aging.

### 1.1 Biological aging and its measurement

Biological aging is a multidimensional progressive accumulation of molecular, cellular, physiological, and functional changes that reduce homeostatic capacity and increase susceptibility to disease and mortality. Unlike chronological age, biological aging captures inter-individual variation in the functioning of biological systems. Consequently, individuals of the same chronological age may differ substantially in terms of their biological age and health risks (4–6). This multidimensional process involves interconnected changes in genomic stability, epigenetic regulation, cellular senescence, mitochondrial function, proteostasis, nutrient sensing, immune regulation, and communication between intercellular cells (7, 8). Biological age is, therefore, best conceptualized as a latent construct estimated using biomarkers rather than a directly observable characteristic.

Epigenetic aging is one approach for estimating biological aging. Epigenetic clocks use DNA methylation (DNAm) patterns at selected cytosine–phosphate–guanine (CpG) sites to estimate chronological age or predicted age-related phenotypes (9, 10). Epigenetic clocks provide DNA methylation-based estimates of age or aging-related phenotypes. Deviations between estimated and expected age have been investigated as biomarkers of biological aging, although their interpretation varies according to the clock, tissue, developmental stage, and outcome. The differences between an individual's DNAm-predicted epigenetic age and chronological age can be described as epigenetic age deviation. When this deviation is operationalized as the residual difference between DNAm-predicted age and chronological age, after accounting for chronological age, it is commonly referred to as epigenetic age acceleration (EAA). Thus, EAA represents an approach to quantify epigenetic age deviation (9, 11).

Early epigenetic clocks, including the Horvath and Hannum clocks, were primarily developed to estimate chronological age (105, 106). Subsequent studies commonly derived measures of EAA from these clocks to quantify deviations between estimated epigenetic age and chronological age or an appropriate reference population (9, 11). Later generation clocks were developed to capture additional aging-related phenotypes, including phenotypic age (PhenoAge), mortality risk (GrimAge), and pace of aging (DunedinPACE) (12–14). These measures should not be considered interchangeable; PhenoAge and GrimAge are trained on phenotypic or mortality-related outcomes, whereas DunedinPACE estimates the pace of biological aging rather than the deviation between estimated and chronological age. The Pediatric-Buccal Epigenetic (PedBE) clock was specifically developed to estimate the epigenetic age of children and adolescents using buccal epithelial cells (15). Accordingly, studies of epigenetic age and epigenetic aging-related measures may use different clocks and age-deviation measures, which are not necessarily interchangeable, and their associations with environmental exposures and health outcomes may not be consistent. A systematic review of 299 publications demonstrated substantial heterogeneity across clocks and outcomes, reinforcing that epigenetic aging is not a uniform construct (16).

In adults, accelerated epigenetic aging has been associated with morbidity, frailty, cognitive and physical functioning, depression, and mortality, although associations vary depending on the epigenetic clock, tissue, population, and outcome examined (9, 13, 17, 18). Epigenetic age acceleration is also associated with potentially modifiable behavioral, environmental, socioeconomic, and psychosocial exposures, including smoking, adiposity, metabolic dysfunction, inflammation, environmental pollutants, and socioeconomic disadvantage (19–22). Physical activity, along with other favorable lifestyle and socioeconomic factors, has been associated with more favorable epigenetic aging profiles, although findings vary across different populations and clocks (23–26).

Early life psychosocial adversity may represent an important pathway linking social exposures to chronic stress. Cumulative adversity may influence biological aging through neuroendocrine, inflammatory, metabolic, behavioral, and epigenetic pathways (27). Evidence linking adverse childhood experiences (ACEs) to epigenetic aging in adults remains inconsistent. A recent systematic review reported generally positive associations between ACE exposure and EAA, including evidence suggestive of a dose–response relationship; however, the literature was concentrated largely in European populations (28). In contrast, a broader systematic review and meta-analysis found no significant pooled association between cumulative ACE exposure and EAA, with substantial heterogeneity across clocks, ACE measures, populations, and analytical approaches (29). Together, these findings suggest that childhood adversity may contribute to epigenetic aging in adults; however, the magnitude and consistency of this association remain unclear and may depend on how adversity and epigenetic aging are measured.

### 1.2 Biological aging during childhood and development

Although biological aging has been studied extensively in adults, its interpretation during fetal development and childhood presents distinct conceptual and methodological challenges. Development and aging involve overlapping processes, including changes in cellular function, epigenetic regulation, immune activity, metabolism, and tissue composition. However, these processes occur alongside rapid growth, cellular proliferation, differentiation, organ maturation, and neurodevelopment in early life. Consequently, epigenetic age deviations observed during gestation or childhood may reflect differences in developmental maturity or programming rather than accelerated aging in the conventional adult sense (30).

This distinction is particularly relevant because epigenetic clocks used in pediatric research have been developed for different developmental stages and purposes. Gestational age clocks, such as the Knight and Bohlin clocks, estimate gestational age from DNAm patterns in perinatal tissues. The difference between DNAm-estimated and clinically estimated gestational age can be expressed as gestational epigenetic age deviation and may be operationalized as gestational epigenetic age acceleration (GEAA). However, in fetal and neonatal populations, this measure may reflect variation in developmental timing or maturity rather than accelerated aging in the conventional adult sense (31, 32). Similarly, chronological age clocks, including the PedBE clock, have been developed to capture age-related methylation patterns during childhood (15). A systematic review of 68 pediatric studies demonstrated the increasing use of epigenetic clocks to examine the relationships between early-life exposures, development, behavior, and health, but also substantial heterogeneity in clocks, tissues, and outcomes examined (33). Thus, the interpretation of epigenetic age estimates and age-deviation measures should be guided by the developmental stage, tissue type, and purpose for which each clock was developed (30).

The interpretation of epigenetic age deviation may change across the life course. During gestation and early infancy, deviations from the expected epigenetic age may primarily reflect developmental timing, fetal maturity, or prenatal programming, whereas during childhood and adolescence, they may reflect an interplay between ongoing maturation and environmentally responsive biological processes (9, 30). Accordingly, epigenetic age deviations in pediatric populations should not necessarily be interpreted as evidence of accelerated aging in the same way as in adults.

Evidence that childhood adversity is associated with biological aging-related processes that are detectable before adulthood supports the potential relevance of epigenetic age deviation and other epigenetic aging-related measures in development. Childhood threat and deprivation have been associated with indicators of biological development and aging, including pubertal timing, telomere length, and DNAm-based measures, and the associations may vary by type of adversity and biological measure (34). More recent longitudinal research has reported associations between early-life adversity and epigenetic aging during the peripubertal period, including associations with subsequent behavioral and mental health outcomes (35). These findings suggest that epigenetic measures may provide a means of investigating how early experiences are biologically embedded during development. However, they did not establish that deviations in gestational or pediatric EAA represent the same aging process observed in adulthood.

The interpretation of epigenetic age deviations in pediatric populations is further complicated by methodological variations. Epigenetic clocks have been developed and validated for specific populations, tissues, age ranges, and outcome definitions, and their performance may vary across developmental stages and populations. DNAm is strongly dependent on tissue and cell types. Estimates may vary according to the methylation platform, preprocessing procedures, statistical model, and reference population (30). These considerations are particularly important when comparing epigenetic aging across different demographic groups. Apparent differences by sex, race, ethnicity, or socioeconomic circumstances may reflect variations in environmental exposures, structural conditions, cellular composition, or clock performance rather than inherent biological differences. This is especially relevant to studies of maternal adversity and offspring biological aging, where social and environmental conditions may influence both exposure patterns and the biological measures used to assess their effects.

Taken together, epigenetic aging measures should be interpreted as developmentally contextualized biomarkers rather than uniform indicators of biological aging across the life course. In the context of maternal adverse childhood experiences (ACEs), associations with offspring epigenetic age may reflect altered fetal development, persistent biological programming, changes in developmental trajectories, and emerging aging-related processes. Determining whether such associations are detectable at birth, persist throughout childhood, or become more closely aligned with conventional measures of biological aging later in life may provide insight into the potential intergenerational consequences of early-life adversity. However, evidence linking maternal ACEs to offspring epigenetic age deviation remains limited, and the developmental significance of the observed deviations in epigenetic aging is not yet well established.

### 1.3 Adverse childhood experiences and intergenerational biological embedding

ACEs are potentially traumatic events occurring before 18 years of age, including abuse, neglect, and household dysfunction, such as caregiver substance use, mental illness, intimate partner violence, incarceration, and chronic instability (36, 37). ACE exposure is associated with a broad range of psychological, behavioral, and physical health outcomes across the life course (37–39). Increasing attention has been focused on potential intergenerational effects, including whether maternal exposure to childhood adversity influences offspring development and health (40–42).

Several plausible but not yet fully established pathways may link maternal ACEs with epigenetic age deviation and other offspring epigenetic aging-related biomarkers. Potential pathways are diverse and may involve genetic and epigenetic mechanisms, maternal hormonal and neurobiological changes, as well as alterations in the intrauterine environment (43, 44). Maternal childhood adversity may produce persistent alterations in hypothalamic–pituitary–adrenal axis function, cortisol regulation, immune activity, metabolic health, and behavior, which may influence the intrauterine environment during a subsequent pregnancy (45–47). Altered maternal stress physiology may affect fetal developmental programming through glucocorticoid signaling and epigenetic regulation of stress-responsive genes, including *NR3C1* and *FKBP5* (48, 49). These mechanisms are consistent with the Developmental Origins of Health and Disease (DOHaD) framework, whereby prenatal environmental conditions can produce persistent changes in biological regulation and health (50–52).

The placenta may be an important intermediary between maternal adversity and fetal biological development. As the maternal–fetal interface, it integrates endocrine, immune, metabolic, and environmental signals and regulates fetal exposure to glucocorticoids and other mediators. Maternal stress has been associated with the placental methylation changes in genes involved in glucocorticoid signaling, including *NR3C1* and *FKBP5*, with potential implications for fetal development (53–55). Maternal adversity may also influence the inflammatory and immune pathways that affect placental function, fetal immune development, and offspring DNA methylation (56–58). Oxidative stress and mitochondrial dysfunction are other potential pathways relevant to biological aging, as chronic stress is associated with increased reactive oxygen species, impaired antioxidant capacity, and mitochondrial dysregulation, which may influence DNA methylation, cellular senescence, and other aging-related processes following maternal adversity (7, 59–63). These pathways may be particularly relevant when epigenetic aging deviation or other epigenetic aging-related measures are assessed at birth, although direct evidence linking maternal ACEs to these mechanisms and offspring epigenetic age remains limited.

Behavioral, metabolic, and environmental pathways may further contribute to intergenerational associations. Maternal smoking, diet, physical activity, sleep, socioeconomic conditions, exposure to environmental pollutants, and ongoing psychosocial stress may be associated with both maternal adversity and offspring biological outcomes (64, 65, 67). These factors may operate alongside maternal physiological pathways and contribute to the observed associations independently or in combination with prenatal biological programming. Their relative importance may vary according to the timing and type of maternal adversity, maternal health during pregnancy, offspring characteristics, and postnatal environment.

Despite evidence linking childhood adversity with biological aging in adults and emerging evidence linking early-life adversity with epigenetic aging in children, significantly less is known about whether maternal childhood adversity is associated with epigenetic aging-related biomarkers in offspring. Developmental timing is particularly important for interpreting such associations. Epigenetic age differences detected in cord blood may reflect prenatal developmental timing or fetal programming, whereas associations observed later in childhood may indicate the persistence or emergence of biological differences following birth (30). Longitudinal studies spanning developmental periods are, therefore, needed to determine whether early epigenetic age deviations persist, attenuate, or become more closely associated with health-relevant biological aging.

Evidence examining the relationship between maternal ACEs and offspring epigenetic age deviations remains sparse. To characterize this emerging literature, a scoping review was conducted to map and synthesize evidence examining the associations between maternal ACEs and offspring epigenetic age deviation and related epigenetic aging measures from birth to 18 years of age. This scoping review will characterize the variation according to developmental stage, type of maternal adversity, offspring characteristics, tissue type, epigenetic clock, and operationalization of epigenetic age deviation, with the aim of identifying key knowledge gaps and priorities for future research.

## 2 Methods

The scoping review was conducted using the methodological framework proposed by Arksey and O’Malley (66). This framework consists of five key stages: (1) identifying the research question, (2) identifying relevant studies, (3) selecting studies, (4) charting the data, and (5) collating, summarizing, and reporting the findings. The research question guiding this review was: “Are maternal adverse childhood experiences (ACEs) associated with epigenetic biomarkers of aging in offspring?” This research aimed to examine whether maternal exposure to early-life adversity is associated with offspring epigenetic age or age deviation measures derived from DNAm-based epigenetic clocks, including EAA and GEAA, and other validated measures, such as pace of aging measures.

Studies using candidate gene methylation analyses were excluded because they did not quantify epigenetic age deviation or other measures of epigenetic aging using validated epigenetic clock algorithms. Candidate gene approaches assess DNA methylation at a limited number of pre-selected individual loci, whereas epigenetic clocks are predictive biomarkers that estimate epigenetic age or aging-related phenotypes using methylation patterns across numerous age-related CpG sites selected from the genome. Consequently, candidate gene studies cannot generate standardized measures of epigenetic age or epigenetic age deviation, which are derived by comparing epigenetic age estimates with the chronological age. Moreover, the biological interpretation of methylation at individual candidate genes fundamentally differs from that of epigenetic clock-derived measures of aging. Restricting the review to studies employing validated DNAm clocks ensured methodological consistency and comparability across studies while improving the interpretability of the review findings.

Prior to commencing the literature search, a university librarian was consulted to refine the search strategy and terms and ensure comprehensive coverage of relevant databases. A comprehensive literature search was performed across six online research databases: MEDLINE (Ovid), EMBASE (Ovid), APA PsycINFO (Ovid), CINAHL (EBSCO), Scopus (Elsevier), and ProQuest Dissertations and Theses Global. The research strategy included keywords related to ACEs in mothers and EAA in children, combined with the Boolean operators AND and OR (see Supplementary Materials). After identifying five highly relevant seed papers that aligned with the research question and met predefined inclusion and/or exclusion criteria, a detailed analysis of their titles, abstracts, and indexed terms was conducted to determine the most appropriate combination of keywords and subject headings for the search strategy (67–71). The initial searches were conducted in July 2025 (see Supplementary Materials for the dates). A total of 1,467 records were identified and imported into the systematic review software Covidence. After excluding 93 duplicates, 1,374 articles remained and were reviewed based on their titles and abstracts. A follow-up search of all databases was conducted on 28 April 2026, to determine whether any new papers relevant to this review had been published since the initial search. In total, 67 new studies were identified and underwent title and abstract reviews. After excluding 93 duplicates, 1441 studies remained and their titles and abstracts were reviewed.

The PECO (Population/Patient/Problem, Exposure, Comparator, and Outcome) framework (72), which is similar to the PICO framework (73), was applied to clearly define the review's inclusion and exclusion criteria. In this review, the PECO elements helped specify the population of interest (pregnant individuals with ACE exposure and their offspring), the exposure (maternal ACEs), and the outcome (child EAA/GEAA) (72). Integrating the PECO framework ensured a systematic approach to selecting and interpreting evidence. Predefined eligibility criteria were established for the scoping review to guide study selection and ensure the inclusion of relevant articles (see Table 1).

**Table 1:** Adapted PECO (population/patient/problem, exposure, comparator, and outcome) framework used to define inclusion and exclusion criteria for the scoping review.

Adapted PECO framework	Inclusion criteria	Exclusion criteria
Population	Offspring (0–18 years of age) of pregnant individuals with self-reported adverse childhood experiences (ACEs) Human study	Offspring over the age of 18 Non-human studies (animal models, using cell lines, or experimentally manipulating cells cultured *ex vivo*)
Exposure	Examined epigenetic biomarkers of aging, including epigenetic age deviation or other measures of epigenetic aging in children using validated DNAm clock(s) Pregnant individuals of offspring (0–18 years of age) with self-reported adverse childhood experience (ACE) data Pregnant or postpartum individuals with available self-reported ACEs data collected during pregnancy or within three years of childbirth ACEs assessed using a valid and reliable questionnaire [e.g., (77)] Included singleton live births only Informed consent obtained from participants for biospecimen collection and genomic analysis Ethical approval for biospecimen collection and genomic analysis	Did not examine epigenetic biomarker aging using validated DNAm clock(s); epigenome-wide association studies Maternal ACEs not self-reported Pregnant or postpartum women without self-reported ACE data; women who were not pregnant or within three years postpartum at the time of the ACE data was collected; the time of collection of maternal ACE data unclear Maternal ACEs assessed using a questionnaire that is not valid Included twins/multiples; did not examine the singleton live birth data separately Lack of participant-informed consent Unclear if ethical approval obtained for the biospecimen and genomic analysis
Outcome	Biospecimens collected from offspring under 18 years of age DNA methylation analysis conducted using biospecimens collected from the offspring Acceptable tissue sources from offspring included peripheral blood, umbilical cord blood, saliva, and buccal epithelial cells Offspring EAA, GEAA, and other validated measures of epigenetic age deviation derived from DNAm-based epigenetic clocks	Biospecimens from offspring aged 18 years or older or age not reported No DNA methylation data reported No tissues analyzed from offspring (e.g., only maternal samples analyzed) No data on offspring EAA, GEAA, or other validated measures of epigenetic age deviation derived from DNAm-based epigenetic clocks
Study characteristics	Studies conducted in any country Studies published in English Timeframe of the study reported Quantitative or mixed-methods studies, cohort studies, cross-sectional studies, longitudinal studies, randomized controlled trials, non-randomized trials, and reviews, including systematic reviews, scoping reviews, and narrative reviews that synthesize original findings Gray literature (e.g., dissertations, theses, conference proceedings) that presents original data	Published in a language other than English Timeframe of the study not reported Theoretical, opinion, or conceptual papers without empirical data Methodologies outside the accepted scope (e.g., commentaries, letters, editorials). Reviews that did not synthesize original data or were not systematic/scoping/narrative in nature Gray literature lacking original data (e.g., abstract-only, conceptual theses)

Article screening was performed by three independent reviewers (DC, MHS, and JC) who completed a calibration exercise based on the inclusion and exclusion criteria to ensure consistency in applying the criteria. The inter-rater agreement score generated by this calibration exercise was 90%, indicating a high level of agreement. After title and abstract screening of the 1,441 articles, the reviewers identified 38 articles for full-text review. After the full-text review, four studies were identified that met the inclusion criteria (see Figure 1). Relevant data from these studies were systematically extracted using a standardized data charting form to ensure consistency and accuracy across the included articles. The key variables extracted included study characteristics, including authors, publication year, country of origin, and study design, as well as population details, such as maternal age, child age, and sample size. Information related to maternal ACEs was also charted, including the type of adversity assessed, severity or cumulative burden of ACE exposure, and the assessment tools used to measure childhood adversity. In addition, detailed information regarding EAA/GEAA assessment methods was collected, including the type of epigenetic clock(s) utilized, tissue type, timing of biological sample collection, and analytical techniques used to estimate epigenetic age deviation. Information on the study design, sample size, statistical analysis methods, and key findings was also extracted. This process allowed us to examine how the associations between maternal ACEs and child EAA/GEAA had been investigated in the research literature.

**Figure 1:**
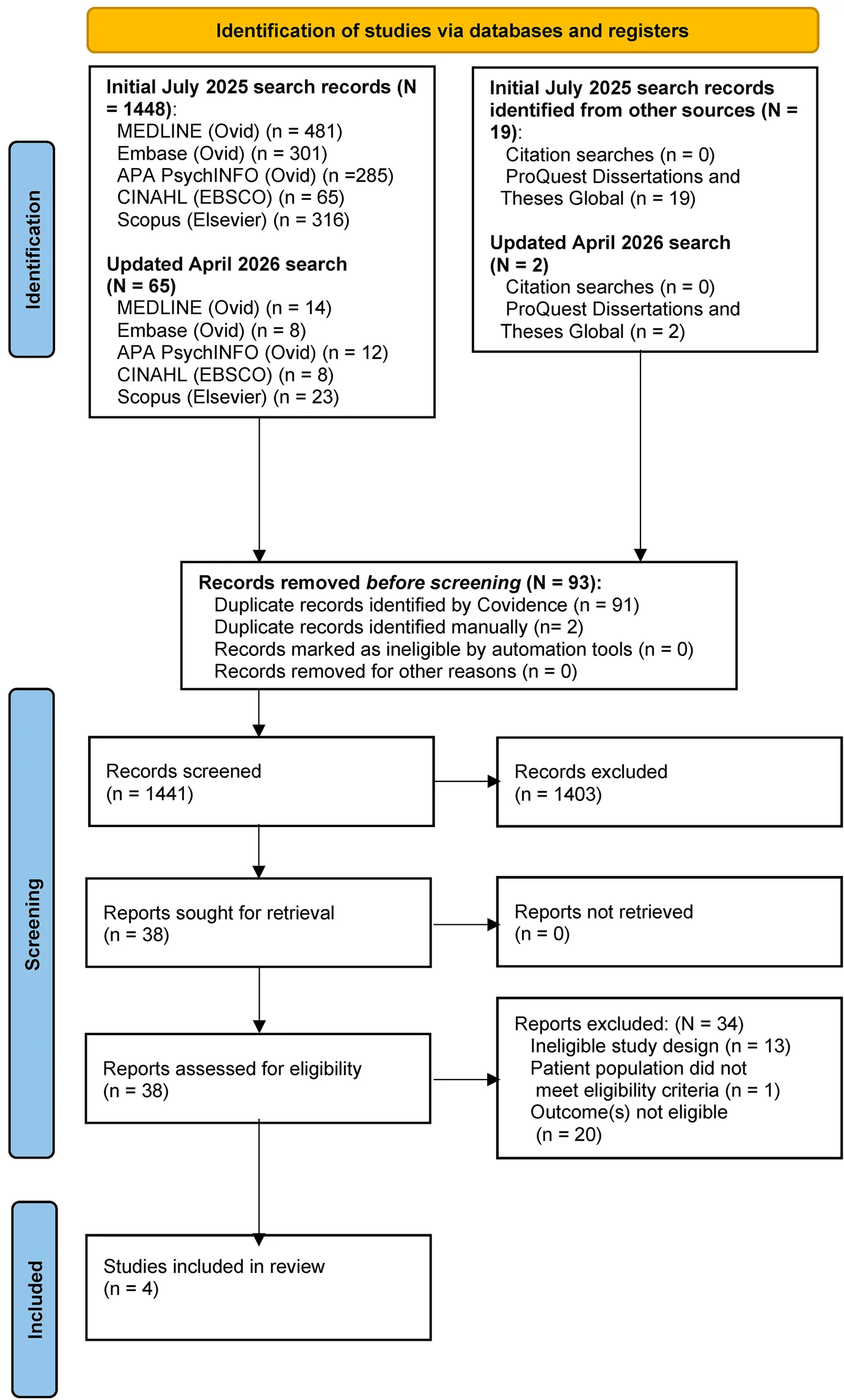
PRISMA flow diagram for scoping review (PRISMA-ScR), adapted from the PRISMA 2020 statement. Source: Page et al. (107). This work is licensed under CC BY 4.0. To view a copy of this license, visit https://creativecommons.org/licenses/by/4.0/. Changes were made to the figure to show the study flow.

The methodological quality of the studies included in this scoping review was assessed using the JBI Assessment of Risk of Bias Tool (74). Two independent reviewers (DC and GEM) conducted the appraisal. Any discrepancies between the reviewers were resolved through discussion. The JBI assessment tool was used to evaluate the risk of bias, adequacy of the study design, and clarity of reporting for each included study. The results of this critical appraisal were used to inform the synthesis of findings.

## 3 Results

### 3.1 Characteristics of the research studies

The four studies that met our inclusion criteria were published between 2024 and 2025 (see Table 2). Three were conducted in the United States (64, 65, 75), and one was conducted in the United Kingdom (68). These studies employed observational designs; three were longitudinal prospective cohort studies in which maternal ACEs were assessed during pregnancy and epigenetic age deviation, and other epigenetic age-related measures were assessed in cord blood or child blood (64, 65, 75). Dye et al. (68) used a retrospective cross-sectional design to examine data from the Accessible Resource for Integrated Epigenomic Studies substudy of the Avon Longitudinal Study of Parents and Children. For this study, women provided retrospective self-reports of their ACEs between 18 and 32 weeks of gestation, when their child was aged approximately 3 years, through mail-in questionnaires, and cord blood epigenetic clocks were used to assess GEAA (68).

**Table 2:** Characteristics of the included studies.

Author(s), year, country	Study design	Sample size (*n*)	Mean maternal age at delivery (years), Standard deviation (SD)	Mean child age (years)	Time of EAA/GEAA assessment	Measures of maternal ACEs	Method used to measure EAA/GEAA (tissue, array, clocks)	Key finding(s)
Dye et al. (2024) (68), UK	Cross-sectional [Accessible Resource for Integrated Epigenomic Studies (ARIES) substudy of the Avon Longitudinal Study of Parents and Children (ALSPAC)]	883 dyads	Mean 29.8 (SD 4.3)	Newborns	Birth	10-item ACEs questionnaiire (76)	Tissue: Cord blood Array: Illumina Infinium Methylation 450 BeadChip Clocks: Knight, Bohlin	Maternal total ACEs were not associated with newborn epigenetic age deviation Sex-specific effects of individual ACE score components: In males, maternal exposure to any type of neglect (*β* = 0.58, 95%CI: 0.05, 1.11, *P* = 0.03) or emotional neglect (*β* = 0.62, 95%CI: 0.09, 1.15, *P* = 0.02) was associated with higher Knight GEAA In females, maternal exposure to parental separation/death (*β* = −0.45, 95%CI: −0.91, 0.00, *P* = 0.05) or parental domestic violence (*β* = −0.67, 95%CI: −1.25, −0.08, *P* = 0.03) was associated with a lower Knight GEAA
Hamlat et al. (2025) (64), USA	Longitudinal cohort [National Heart, Lung, and Blood Institute Growth and Health Study (NGHS)]	215 dyads	Mean 39.2 (SD 1.1)	Mean 8.3 years (range: 2–17)	Childhood/adolescence	Stress and Adversity Inventory (STRAIN) + Felitti items (physical and sexual abuse; substance abuse) (76, 78)	Tissue: Saliva Array: Illumina Infinium Methylation 450 BeadChip Clocks: Horvath, DunedinPACE	Significant difference in Horvath clock EAA in children of Black pregnant individuals compared to children of White pregnant individuals [t(213) = 1.68, *P* < 0.05, d = 0.23]; children of Black pregnant individuals were approximately 4 months younger epigenetically than their chronological age, whereas children of White pregnant individuals were approximately 6 weeks older epigenetically No significant difference in the epigenetic pace of aging for the children of Black pregnant individuals compared to the children of White pregnant individuals [t(213) = 0.26, *P* = 0.80, d = 0.04] Maternal general abuse and sexual abuse were associated with accelerated Horvath EAA in Black children only (abuse < 13: b = 0.81; physical abuse: b = 1.69); no effect in White children No association between maternal abuse and pace of aging
McKenna et al. (2024) (75), USA	Prospective cohort: (Pregnancy Study; Maternal Stress and Infant Microbiome Study; Echo Study)	80 dyads	Mean 24.5 (range: 18–39)	Mean 3.04 (range: 1.96–4)	Birth or at ∼41-weeks equivalent gestational age	10 item ACEs questionnaire + Child Trauma Questionnaire (CTQ) - short form (emotional, physical, sexual abuse) (76, 77)	Tissue: Peripheral blood (spots/clots) Array: Illumina Infinium Methylation EPIC BeadChip Clock: Knight	No significant association between maternal ACEs and neonatal GEAA Higher infant Knight GEAA buffered the effect of maternal sexual abuse on children's internalizing (*β* = −0.31, *P* = .004) and externalizing (*β* = −0.28, *P* = 0.01) behavioral symptoms
Sosnowski et al. (2024) (65), USA	Prospective cohort (SHIP, PREDICT) [Stress and Health in Pregnancy (SHIP); Prospective Research on Early Determinants of Illness and Children's Health Trajectories (PRIEDICT)]	332 dyads	Mean 29.0 (SD 5.7)	Newborns	Birth	10-item ACEs questionnaire (76)	Tissue: cord blood Array: Illumina Infinium Methylation EPIC BeadChip Clocks: PedBE, Knight, Bohlin, EPIC GA, SHIP-PREDICT	Maternal ACEs showed no direct main effects on any of the five epigenetic clocks Association emerged via interaction with maternal restless sleep during pregnancy (b = 1.04, se = 0.51, *P* = 0.04); newborns of mothers with both ACE exposure and moderate-to-severe restless sleep showed accelerated EPIC GA GEAA (mean EPIC GA acceleration = 1.04, 95% CI = 0.25, 1.83)

Maternal ACEs were primarily assessed through retrospective self-reports collected during the prenatal period, typically between 18 and 32 weeks of gestation (65, 68, 75). Offspring DNAm was profiled at various developmental time points, including at birth or shortly after birth (65, 68, 75) or during childhood and adolescence (64).

Various tissue types were used in the epigenetic clock analyses, including umbilical cord blood (65, 68), peripheral blood (75), and saliva (64). All four studies measured DNAm at a single time point and adjusted for several covariates, including offspring sex, race, maternal BMI, and pregnancy complications (64, 65, 68, 75). The sample sizes of the maternal-child dyads ranged from 80 (68) to 883 (75). The mean maternal age at delivery varied from 24.5 (75) to 39.2 years (64). The mean age of the children at the time of DNA sample collection ranged from birth (65, 68) to 8.3 years (64). All studies included White participants of European ancestry. Hamlat et al. (64) also included Black maternal-child dyads and compared them to White maternal-child dyads to investigate whether racial background influenced the association between maternal childhood adversity and offspring epigenetic age deviation.

Maternal ACEs were assessed using validated instruments, including adaptations of the original ACEs questionnaire developed by Felitti et al. (76), the Childhood Trauma Questionnaire (CTQ) (77), and the Stress and Adversity Inventory (STRAIN) (78). All studies relied on maternal self-reports to capture data on early maternal adversity.

Offspring DNAm was profiled using the Illumina Infinium HumanMethylation450 (450K) (64, 68) or MethylationEPIC (850K) BeadChip (65, 75). Epigenetic age deviation was assessed using multiple clocks, including the Horvath Pan-Tissue and PedBE clocks, while gestational epigenetic age deviation was assessed using gestational-age clocks, including Knight, Bohlin, EPIC GA, and SHIP-PREDICT. DunedinPACE was additionally used as a measure of the pace of biological aging. The tissue types used to determine epigenetic aging include umbilical cord blood (65, 68), peripheral blood (75), and saliva (64).

### 3.2 Associations between maternal ACEs and offspring epigenetic age deviation and aging-related measures

Maternal ACEs were not consistently associated with offspring epigenetic age deviation or other epigenetic aging-related measures (see Table 2). Studies have reported null main effects between cumulative maternal ACE scores and child epigenetic aging; however, associations were noted in subgroup analyses. Hamlat et al. (64) found that maternal childhood physical and sexual abuse predicted increased Horvath EAA in Black children, with physical abuse associated with a 1.69-year increase and sexual abuse with a 1.17-year increase. No significant associations were observed in White children. Similarly, Sosnowski et al. (65) reported no main effects of maternal ACEs on epigenetic aging but identified a significant interaction whereby increased newborn GEAA (mean EPIC GA acceleration = 1.04) occurred only when mothers experienced both ACEs and restless sleep during pregnancy. Dye et al. (68) found that total maternal ACEs scores were not associated with newborn GEAA; however, significant associations emerged in subgroup analyses. Specifically, maternal exposure to any type of neglect (*β* = 0.58, 95%CI: 0.05–1.11, *P* = 0.03) or emotional neglect (*β* = 0.62, 95%CI: 0.09–1.15, *P* = 0.02) was associated with higher Knight GEAA among male newborns, whereas maternal exposure to parental separation (*β* = −0.45, 95%CI: −0.91–0.00, *P* = 0.05) or parental domestic violence (*β* = 0.67, 95%CI: −1.25 to −0.08, *P* = 0.03) was associated with lower Knight GEAA among female newborns. McKenna et al. (75) did not report a significant association between maternal childhood adversity and neonate EAA; however, neonate-accelerated epigenetic aging was found to attenuate the impact of maternal childhood sexual abuse on child internalizing (*β* = −0.31, *P* = .004) and externalizing (*β* = −0.28, *P* = 0.01) behavioral symptoms.

Maternal mental health, particularly prenatal depression and anxiety, was examined as a potential factor that may influence the relationship between maternal ACEs and EAA. Dye et al. (68) reported that these symptoms did not statistically mediate the association between maternal ACEs and newborn epigenetic age deviation.

### 3.3 Quality assessment

Overall, the studies included in this scoping review demonstrated high methodological quality and low risk of bias, as determined by two independent researchers (DC and GEM) who reached 100% agreement on the assessment of the studies using the JBI Assessment of Risk of Bias tool (see Table 3) (74). A primary strength was the use of prospective longitudinal cohort designs in three of the four studies (64, 65, 75), which allowed for a more robust evaluation of the relationship between maternal ACEs and offspring biological aging. All studies employed validated measures of childhood adversity and incorporated a wide range of epigenetic aging biomarkers, including seven distinct epigenetic clocks as indicators of biological or gestational aging. Biospecimens comprised various tissue types, including cord blood, peripheral blood, and saliva, enhancing the breadth of biological assessment across studies.

**Table 3:** Summary of the quality assessment results.

JBI appraisal Question [Barker et al. (74)]	Dye et al. (68)	McKenna et al. (75)	Sosnowski et al. (65)	Hamlat et al. (64)
1. Inclusion criteria clearly defined?	Yes	Yes	Yes	Yes
2. Objective, standard criteria used?	Yes	Yes	Yes	Yes
3. Exposure measured validly/reliably?	Yes	Yes	Yes	Yes
4. Outcomes measured validly/reliably?	Yes	Yes	Yes	Yes
5. Confounding factors identified?	Yes	Yes	Yes	Yes
6. Strategies for confounding stated?	Yes	Yes	Yes	Yes
7. Appropriate statistical analysis?	Yes	Yes	Yes	Yes
8. Subjects and setting described?	Yes	Yes	Yes	Yes
Overall appraisal/inclusion	High	High	High	High

Three studies utilized multivariable statistical models that were adjusted for important maternal and child confounders, such as maternal smoking, pre-pregnancy body mass index, socioeconomic factors, and cell-type composition (65, 68, 75). These adjustments strengthen the confidence that the observed associations were not solely attributable to alternative explanations. However, considerable heterogeneity exists in the epigenetic biomarkers used, making direct comparisons across studies challenging and limiting opportunities for synthesis. Sample sizes varied substantially, ranging from 80 to 883 maternal-child dyads, which may have affected statistical power and contributed to inconsistent findings. The study by McKenna et al. (75) had a smaller sample size of 80 maternal-child dyads, which may be vulnerable to type II error and could have lacked sufficient power to detect modest associations between maternal ACEs and offspring epigenetic age deviation.

## 4 Discussion

This scoping review identified four studies that examined the association between maternal adverse childhood experiences (ACEs) and epigenetic biomarkers of aging in pediatric populations that met our inclusion criteria, highlighting the limited evidence currently available. Three of the four studies were prospective longitudinal cohorts and reported that the associations between maternal ACEs and offspring epigenetic age deviation varied according to factors such as offspring sex, maternal sleep quality during pregnancy, and race (64, 65, 68). McKenna et al. (75) found no direct association between maternal adversity and GEAA, although maternal adversity moderated the relationship between ACEs and children's behavioral outcomes. Overall, the findings suggest that maternal ACEs may be associated with offspring epigenetic age deviation or other epigenetic aging-related measures; however, these associations are complex, context-dependent, and supported by a limited body of evidence.

Across the included studies, the associations between maternal ACEs and offspring epigenetic age deviation appeared to be highly context-dependent. Offspring sex, maternal race, maternal sleep quality during pregnancy, and the specific type of adversity experienced modified the observed relationships. Dye et al. (68) reported sex-specific associations: maternal neglect predicted accelerated GEAA in males, whereas parental separation or domestic violence were associated with decreased GEAA in females. Similarly, Hamlat et al. (64) found significant associations only among Black maternal-child dyads, while Sosnowski et al. (65) observed effects only when maternal ACEs co-occurred with moderate-to-severe restless sleep during pregnancy. Given the limited body of evidence and heterogeneity in sample characteristics and methodologies of the included studies, it is recommended that these potential contextual differences be interpreted cautiously. It is important to note that epigenetic clocks are imperfect biomarkers that are subject to biases based on the training datasets used in their creation. Prediction models represented by epigenetic clocks can underperform in certain groups based on sociodemographic factors, such as sex/gender and race, which are underrepresented in their training datasets (79–81). There is likely an inadequate conceptualization of these sociodemographic dimensions in the creation of epigenetic clocks, although this varies between clocks, as newer clocks are more responsive to social inequalities, which could contribute to inaccuracies or inequalities in epigenetic deviation metrics. Collectively, the findings from the included studies suggest that maternal childhood adversity is unlikely to influence offspring epigenetic aging uniformly and instead interacts with biological, social, and prenatal environmental factors.

Maternal ACEs showed consistent sex-specific associations with offspring epigenetic age deviation. Dye et al. (68) reported that maternal ACEs were associated with accelerated GEAA in male newborns but not in females. In males, maternal experiences of neglect, particularly emotional neglect, and parental substance abuse were associated with accelerated GEAA, whereas in females, maternal experiences of parental separation/death or domestic violence were associated with decelerated GEAA. These findings are consistent with evidence, suggesting that male fetuses may be more vulnerable to prenatal adversity and its effects on epigenetic programming and developmental trajectories (82–84). Similarly, McGill et al. (85) found that prenatal maternal anxiety was associated with accelerated epigenetic aging in children, with males showing greater sensitivity, and that accelerated epigenetic aging was associated with increased externalizing behaviors between 6 and 10 years of age. In contrast, the lower GEAA observed among females may reflect sex-specific developmental responses to maternal adversity, potentially shaped by differences in biological responses to maternal childhood trauma and postnatal factors, such as supportive caregiving and nutrition (86–88). Collectively, these findings suggest that offspring sex may modify the biological embedding of maternal adversity through differences in fetal development, placental function, and stress-responsive epigenetic programming (89–91). However, the mechanisms underlying these sex-specific associations remain poorly understood.

The observed associations between maternal ACEs and offspring epigenetic age deviation or aging-related measures varied according to the epigenetic clock used, suggesting that different measures capture distinct aspects of biological aging and developmental processes. This distinction is important because DunedinPACE estimates the pace of biological aging rather than the deviation between DNAm-estimated age and chronological age. Hamlat et al. (64) examined the relationship between maternal childhood adversity and offspring epigenetic age deviation as well as the pace of aging using the Horvath and DunedinPACE measures, respectively. They found significant associations only for Horvath EAA among Black maternal-child dyads, whereas no significant associations were observed using the DunedinPACE. These findings suggest that various epigenetic measures may differ in their sensitivity to maternal adversity and may capture distinct aspects of aging-related biology or developmental programming.

Further evidence supporting this interpretation is provided by Nwanaji-Enwerem et al. (92), who assessed maternal ACEs approximately 18 years after childbirth in the CHAMACOS cohort. Although this study did not meet the inclusion criteria for the present review because maternal ACEs were measured long (nearly two decades) after pregnancy, its findings warrant consideration. Offspring of mothers reporting one to two ACEs exhibited accelerated epigenetic aging, as measured by the Horvath clock and Horvath intrinsic EAA. In contrast, offspring of mothers reporting three or more ACEs did not differ significantly from those whose mothers reported no ACEs. The reasons for these associations are unclear. The authors proposed that the thresholds for detecting associations may differ according to the specific epigenetic biomarker examined and further suggested that chronic exposure to higher levels of adversity may induce physiological adaptations that influence epigenetic aging measures differently (93). However, these hypotheses require further investigation.

More broadly, the interpretation of findings across studies is complicated by the increasing diversity of epigenetic aging measures. First-generation clocks, such as the Horvath and Hannum clocks, were developed to estimate chronological age, whereas second-generation clocks incorporate biomarkers of physiological dysregulation and health risks. In pediatric populations, deviations from the expected gestational or chronological epigenetic age, including GEAA and pediatric-specific clocks such as PedBE, may instead reflect developmental maturity and biological responses to prenatal and early-life exposures rather than aging in the conventional sense. Consequently, differences in the epigenetic clock selection may contribute substantially to inconsistencies across studies and limit direct comparison of findings. Greater methodological consistency in the selection and reporting of epigenetic aging biomarkers is important for improving comparability across future studies and determining which measures are most informative for investigating the intergenerational effects of maternal adversity.

Current evidence suggests that epigenetic age deviation measures, such as EAA, GEAA, and other aging-related molecular biomarkers such as DNAm telomere length, may reflect fetal developmental programming and biological responses to prenatal and early-life environmental exposures rather than aging in the conventional sense (30, 94, 95). Previous studies have linked deviations in pediatric epigenetic age with extreme prematurity, birth weight, and childhood physical and mental health phenotypes (96–98), suggesting that these biomarkers may represent indicators of developmental maturity or altered developmental trajectories. However, the clinical significance of these epigenetic age deviations, particularly across different developmental stages, remains unclear. In adults, accelerated epigenetic aging has consistently been associated with increased morbidity and mortality, frailty, neurodegenerative diseases, depression, and other adverse health outcomes (9, 17, 18, 99–102), whereas decelerated epigenetic aging has been associated with lower all-cause mortality and reduced health risks (103, 104). In pediatric populations, however, the biological interpretation of epigenetic age deviation may vary across developmental stages. In cord blood, GEAA may primarily reflect variations in fetal developmental timing or maturity, whereas EAA measured during later childhood may increasingly capture environmentally responsive biological processes. Consequently, deviations in epigenetic age during fetal development may not have the same biological or clinical implications as those observed during infancy, childhood, adolescence, or adulthood. Determining whether and at what developmental stages EAA predicts subsequent health outcomes and whether it mediates associations between maternal ACEs and later physical, cognitive, behavioral, and mental health outcomes remains an important area for future research. Addressing these questions will require longitudinal studies following maternal-child dyads from pregnancy through childhood and adolescence, and ideally into adulthood. However, the significant methodological, financial, and logistical challenges of establishing and maintaining such prospective cohorts, particularly those capable of assessing intergenerational effects, have contributed to the limited evidence available to date.

### 4.1 Limitations of research literature

This scoping review identified several important limitations in the literature examining the associations between maternal ACEs and offspring epigenetic age deviation and other epigenetic age-related measures. The most significant limitation is the small body of available evidence. Only four studies met the inclusion criteria despite screening 1,441 records, highlighting the limited research in this area. The small number of eligible studies precluded quantitative synthesis and limited the ability to determine whether inconsistencies in findings reflect true biological differences or methodological variation. The limited evidence base also increases the potential influence of publication bias, whereby unpublished studies reporting null or non-significant findings could substantially alter the overall interpretation of the evidence.

Although the included studies were generally of moderate to high methodological quality and employed rigorous statistical adjustments for important confounding variables, the evidence was limited by a lack of longitudinal follow-up. Only one study has examined epigenetic aging markers during childhood and adolescence (64), and few studies have assessed whether the observed epigenetic age alterations persisted across development. Consequently, it remains unclear whether epigenetic age deviations observed at birth or during childhood represent transient developmental differences or persistent biological changes. More importantly, insufficient longitudinal evidence limits our understanding of whether epigenetic age deviations mediate the association between maternal adversity and subsequent physical, behavioral, cognitive, or mental health outcomes in offspring.

Substantial methodological heterogeneity across studies further limits their comparability. The included studies differed in study design, biospecimen type (e.g., umbilical cord blood, peripheral blood, and saliva), timing of sample collection, epigenetic clock selection, and statistical analysis approaches. These differences may contribute to inconsistent findings and complicate the interpretation of studies. In particular, first-generation, second-generation, gestational, and pediatric-specific epigenetic clocks have been developed to capture different aspects of chronological age, age-related phenotypes, developmental timing, and biological aging. Consequently, differences in the observed associations may reflect variations in the underlying biological constructs being measured and differences in the measurement approaches.

The generalizability of the findings was also limited by the characteristics of the study population. Three of the four studies were conducted in the United States, and although some cohorts included participants from diverse racial and ethnic backgrounds, the individual study populations were often relatively modest in size and demographically homogeneous. Limited sample sizes may have reduced the statistical power to detect subtle associations and constrained the ability to examine potential effect modification, including differences according to offspring sex, maternal sociodemographic characteristics, or prenatal environmental exposures. Furthermore, the predominance of US-based cohorts limits the applicability of the findings to populations living in different social, cultural, and healthcare contexts, where structural inequities, social determinants of health, and access to protective resources may influence the biological embedding of adversity.

Another important limitation was the retrospective assessment of maternal ACEs. All included studies relied on maternal self-reports of childhood adversity, which are susceptible to recall bias, under-reporting, and exposure misclassification. Although retrospective ACE measures are widely used in epidemiological research, inaccuracies in reporting may attenuate the observed associations. In addition, several cohorts have reported a relatively low prevalence or limited variability in ACE exposure, which may have reduced the ability to detect dose–response relationships or more subtle associations between maternal adversity and offspring epigenetic age deviation.

Finally, there are important developmental knowledge gaps. Most studies have assessed offspring epigenetic aging either at birth or during later childhood and adolescence, with no eligible studies examining infancy. Only one study included follow-up beyond early childhood, limiting our understanding of how epigenetic age deviations change across developmental stages and whether they are associated with subsequent health outcomes. Further research using larger, geographically diverse longitudinal cohorts with repeated epigenetic assessments is therefore needed to determine the persistence, developmental significance, and clinical relevance of offspring epigenetic age deviations. Until such evidence is available, the role of epigenetic aging in the intergenerational biological embedding of maternal adversity remains uncertain.

## 5 Conclusions

This scoping review identified limited but emerging evidence that maternal ACEs may be associated with epigenetic age deviations or other epigenetic aging-related biomarkers in offspring, providing preliminary evidence consistent with the potential intergenerational biological embedding of early-life adversity. However, this does not establish that maternal ACEs cause accelerated biological aging in offspring. Instead, it provides preliminary evidence of associations with epigenetic age deviation, the developmental meaning of which remains unclear. However, the evidence base remains small, comprising only four eligible studies, and the findings were inconsistent and context-dependent. The associations between maternal ACEs and offspring EAA or GEAA varied according to factors including offspring sex, maternal race, and maternal sleep during pregnancy. Interpretation was further constrained by substantial methodological heterogeneity in the study design, biospecimen type, timing of sample collection, and epigenetic clocks. Moreover, the biological meaning of epigenetic age differences may differ across developmental stages, particularly between fetal life, infancy, childhood, and adolescence.

Future research should prioritize larger, longitudinal studies with greater demographic diversity and methodological consistency, alongside mechanistic investigations of the biological pathways linking maternal adversity with offspring epigenetic aging. Longitudinal designs spanning pregnancy through childhood and adolescence are particularly important for determining whether observed epigenetic age differences persist, attenuate, or emerge during development. Repeated assessment of epigenetic aging alongside physical, behavioral, cognitive, and mental health outcomes will also be critical for establishing whether these biomarkers have prognostic or clinical relevance.

Overall, epigenetic aging deviation and other epigenetic aging-related biomarkers provide a promising framework for investigating the intergenerational biological embedding of maternal childhood adversity. However, further research is needed before epigenetic age differences in offspring can be interpreted as evidence of altered biological aging or used to inform clinical and preventive strategies. Clarifying the developmental, environmental, and mechanistic pathways underlying these associations may ultimately advance our understanding of how early-life adversity is biologically embedded across generations and identify opportunities for early prevention and intervention.
