# Supplementary material for: Maternal adverse childhood experiences and epigenetic aging in pediatric populations: a scoping review
Source: Front Child Adolesc Psychiatry. 2026 Oct 5;5:1913117. doi: 10.3389/frcha.2026.1913117 (PMC13685535; doi:10.3389/frcha.2026.1913117)
Supplement: Supplementary file 1 [file Datasheet1.docx]

**Maternal Adverse Childhood Experiences and Epigenetic Aging in Children: A Scoping Review**

**Debjani Chanda^1^, Munawar Hussain Soomro^2,3^, Gillian England-Mason^2,3^, Jaeden Cole^4^ Deborah Dewey^2,3,4,5^**

**Affiliations:**

^1^Bachelor of Health Sciences Program, Cumming School of Medicine, University of Calgary, Calgary

^2^Department of Pediatrics, Cumming School of Medicine, University of Calgary, Calgary, Alberta, Canada.

^3^Owerko Centre, Alberta Children’s Hospital Research Institute, University of Calgary, Calgary, Alberta, Canad

^4^Medical Sciences Graduate Program, University of Calgary, Calgary, Alberta, Canada

^5^Department of Community Health Sciences, Cumming School of Medicine, University of Calgary, Calgary, Alberta, Canada

^6^Hotchkiss Brain Institute, University of Calgary, Calgary, Alberta, Canada

**Database Search Strategies**

**Table S1: Summary of Database Search Strategies**

|  | MEDLINE (Ovid) | EMBASE (Ovid), | APA PsycINFO (Ovid) | CINAHL (EBSCO), | Scopus (Elsevier) | ProQuest Dissertations and Theses Global |
| --- | --- | --- | --- | --- | --- | --- |
| Search Conducted | 1946 to July 3, 2025 | 1974 to July 3, 2025 | 1806 to June 2025 Week 5 | 1937 to July 9, 2025 | 1788 to July 23, 2025 | 1997 to July 9, 2025 |
| Search String | See below | See below | See below | See below | See below | See below |
| Limits | -English only  -Human | -English only  -Humans | -English only  -Humans | -English  only  -Humans | -English only  -Humans | -English  only  -Humans |

**Ovid MEDLINE(R) ALL <1946 to July 03, 2025>**

1	exp Adverse Childhood Experiences/	5113

2	exp Child Abuse/	36777

3	exp Intimate Partner Violence/ or exp Stress Disorders, Post-Traumatic/ or exp Domestic Violence/	102010

4	exp Poverty/ or exp Child Poverty/	52319

5	exp Discrimination, Psychological/ or exp Disability Discrimination/ or exp Social Discrimination/	39392

6	exp Mental Disorders/	1554866

7	exp Adverse Childhood Experiences/	5113

8	exp Child Abuse/	36777

9	exp Pregnancy/ or exp Mothers/	1103260

10	exp Prenatal Exposure Delayed Effects/ or exp Prenatal Care/ or exp Prenatal Diagnosis/	153920

11	exp Epigenesis, Genetic/ or exp Aging/ or exp DNA Methylation/	516207

12	exp Epigenomics/	9006

13	exp Infant, Newborn/	706567

14	exp Exposure to Violence/ or exp Intimate Partner Violence/ or exp Domestic Violence/ or exp Violence/	121482

15	exp Poverty/ or exp Child Poverty/	52319

16	exp Pregnancy Complications/ or exp Fertility/	546161

17	exp Biological Clocks/	16618

18	exp Birth Weight/	48493

19	exp Child, Preschool/ or exp Child Development/	1076939

20	exp Neurodevelopmental Disorders/ or exp Child Development/	286162

21	exp Social Discrimination/ or exp Disability Discrimination/ or exp Discrimination, Psychological/	39392

22	exp Substance-Related Disorders/ or exp Mental Disorders/ or exp Depression/ or exp Mental Health/	1725643

23	7 or 8 or 14 or 15 or 21 or 22	1890988

24	exp Parents/	156806

25	9 or 10 or 16 or 24	1266050

26	exp Epithelial Cells/	685948

27	11 or 12 or 17 or 26	1190255

28	exp Fetal Blood/	32822

29	13 or 18 or 19 or 20 or 28	1780723

30	23 and 25 and 27 and 29	556

31	limit 30 to (English language and humans)	484

**Embase <1974 to 2025 July 03>**

1	exp childhood adversity/	73220

2	exp child abuse/ or exp domestic violence/	82267

3	exp child poverty/	276

4	Adverse Childhood Experience*.mp.	7773

5	exp childhood trauma/ or exp child abuse/	67093

6	exp mother to child transmission/ or exp expectant mother/ or exp mother fetus relationship/ or exp mother child relation/ or exp mother/	194822

7	exp pregnant woman/ or exp mother/ or exp pregnancy/	1033792

8	exp prenatal exposure/	43362

9	exp acceleration/ or exp epigenetics/ or exp DNA methylation/ or exp aging/	598825

10	Epigenetic Age*.mp.	2145

11	Biological Clock*.mp.	3419

12	exp infant/ or exp infant disease/ or exp infant mortality/	2978017

13	exp birth weight/	177135

14	exp child/	3429133

15	Children*.mp.	1832637

16	exp child development/	54704

17	exp fetus blood/ or exp fetus outcome/ or exp fetus blood sampling/ or exp fetus disease/ or exp fetus mortality/ or exp fetus/ or exp fetus risk/ or exp fetus cell/ or exp fetus development/ or exp fetus weight/	522851

18	exp domestic violence/ or exp family violence/ or exp physical violence/ or exp sexual violence/ or exp violence/ or exp exposure to violence/	196886

19	Household Dysfunction.mp.	488

20	exp social discrimination/ or exp auditory discrimination/ or exp employment discrimination/ or exp speech discrimination/ or exp visual discrimination/	70072

21	1 or 2 or 3 or 4 or 5 or 18 or 19 or 20	283777

22	exp maternal death/ or exp maternal fetal transmission/ or exp maternal exposure/	12193

23	Parent*.mp.	794931

24	6 or 7 or 8 or 22 or 23	1781803

25	Epigenetic Age Acceleration.mp.	827

26	exp DNA methylation/	126772

27	9 or 10 or 11 or 25 or 26	602154

28	exp childhood/	114508

29	exp adolescent disease/ or exp adolescent/ or exp adolescent development/ or exp adolescent health/	2011439

30	12 or 13 or 14 or 15 or 16 or 17 or 28 or 29	6238156

31	21 and 24 and 27 and 30	328

32	limit 31 to (human and English language)	316

**APA PsycInfo <1806 to June 2025 Week 5>**

1	exp Mental Health/ or exp Childhood Development/ or exp Trauma/ or exp Child Abuse/ or Adverse childhood experience*.mp. or exp Child Neglect/ or exp Childhood Adversity/ or exp Adolescent Development/	428844

2	exp Childhood Adversity/	7502

3	exp Child Abuse/	38200

4	exp Childhood Development/ or exp Child Abuse/ or exp Childhood Adversity/ or exp Behaviour Problems/ or exp Stress/ or exp Mental Health/ or exp Dysfunctional Family/	464395

5	exp Expectant Mothers/ or exp Single Mothers/ or exp Mothers/	51615

6	exp Expectant Mothers/ or exp Pregnancy/ or Pregnant Women.mp.	63073

7	exp Physiological Aging/ or exp Aging/ or exp Acceleration Effects/ or exp Epigenetics/ or exp Biological Markers/ or exp Chronological Age/ or Epigenetic Age Acceleration.mp.	125877

8	exp Epigenetics/ or exp DNA/ or exp Gene Expression/ or exp Biological Markers/ or DNA methylation.mp.	44343

9	Biological Clock*.mp.	2536

10	Epigenetic Age*.mp.	183

11	exp Aging/ or exp Physiological Aging/	99412

12	exp Infant Development/ or Infant*.mp.	136775

13	exp Infant Development/ or exp Prenatal Exposure/ or exp Fetus/ or exp Prenatal Development/ or Fetus*.mp. or exp Mother Child Relations/	66630

14	exp Childhood Development/ or exp Early Childhood Development/ or Childhood*.mp.	291528

15	exp Adolescent Development/ or Adolescent.mp. or exp Adolescent Psychology/ or exp Adolescent Psychopathology/ or exp Adolescent Characteristics/ or exp Adolescent Health/	450590

16	exp Birth Weight/	3790

17	1 or 2 or 3 or 4	596216

18	5 or 6	105588

19	7 or 8 or 9 or 10 or 11	145677

20	12 or 13 or 14 or 15 or 16	790173

21	17 and 18 and 19 and 20	304

22	limit 21 to (human and English language)	287

**CINAHL**

EBSCO Export

| **S#** | S7 |
| --- | --- |
| **Query (user-entered)** | (("maternal adverse childhood experience*" OR "maternal ACEs" OR "maternal childhood trauma" OR "maternal early life adversity" OR “adverse childhood experience*” OR “ACE*”)) AND (("epigenetic age acceleration" OR "epigenetic clock" OR "biological aging" OR "DNA methylation" OR “biological age”)) AND ((child* OR infant* OR offspring OR newborn OR neonate* OR adolescent* OR teenager)) |
| **Query (expanded/display term)** | (("maternal adverse childhood experience*" OR "maternal ACEs" OR "maternal childhood trauma" OR "maternal early life adversity" OR “adverse childhood experience*” OR “ACE*”)) AND (("epigenetic age acceleration" OR "epigenetic clock" OR "biological aging" OR "DNA methylation" OR “biological age”)) AND ((child* OR infant* OR offspring OR newborn OR neonate* OR adolescent* OR teenager)) |
| **Search run Date and Time** | 2025-07-09T00:32:02.287Z |
| **Results (count)** | 66 |
| **Search Mode** | Find all my search terms. |
| **Interface** | EBSCOhost Research Databases |
| **Database(s)** | CINAHL Plus with Full Text |

**Scopus (Elsevier)**

EXPORT DATE: 23 July 2025

Hamlat E.J., Mayer S.E., Laraia B., Moffitt T.E., Surachman A., Dutcher E.G., Zhang J., Lu A.T., Yang T.T., Mashash M., Slavich G.M., Epel E.S.

AUTHOR FULL NAMES: Hamlat, Elissa J. (55488555300); Mayer, Stefanie E. (56134955500); Laraia, Barbara (6603394700); Moffitt, Terrie E. (58146117700); Surachman, Agus (56707348600); Dutcher, Ethan G. (57205486419); Zhang, Joshua (57221387969); Lu, Ake T. (57201815586); Yang, Tony T. (7404656399); Mashash, Meital (57211079262); Slavich, George M. (16023003700); Epel, Elissa S. (35416705700)

55488555300; 56134955500; 6603394700; 58146117700; 56707348600; 57205486419; 57221387969; 57201815586; 7404656399; 57211079262; 16023003700; 35416705700

Maternal Childhood Adversity Accelerates Epigenetic Aging of Children

(2025) Health Psychology, 44 (5), pp. 479 - 488, Cited 1 times.

DOI: 10.1037/hea0001427

https://www.scopus.com/inward/record.uri?eid=2-s2.0-105003620888&doi=10.1037%2fhea0001427&partnerID=40&md5=20dcee5766f5771553efbe3988a8be76

DOCUMENT TYPE: Article

PUBLICATION STAGE: Final

SOURCE: Scopus

Guleroglu F.Y., Cetin A., Coskun G.P., Caliskan M., Karaduman F., Bilginer C., Misirlioglu R., Tekin S., Al M.N., Caklili T., Tutar Y.

AUTHOR FULL NAMES: Guleroglu, Filiz Yarsilikal (40461412700); Cetin, Ali (35571650500); Coskun, Goknil Pelin (56872719000); Caliskan, Meltem (59416488300); Karaduman, Fulya (59468618300); Bilginer, Can (59011625300); Misirlioglu, Resat (59468783400); Tekin, Sinem (58987377200); Al, Merve Nur (59537465500); Caklili, Tugce (59468618400); Tutar, Yusuf (12767764000)

40461412700; 35571650500; 56872719000; 59416488300; 59468618300; 59011625300; 59468783400; 58987377200; 59537465500; 59468618400; 12767764000

The role of 1-Deoxysphingolipids and Polyamines in the pathogenesis of placental syndrome

(2025) BMC Pregnancy and Childbirth, 25 (1), art. no. 51, Cited 1 times.

DOI: 10.1186/s12884-025-07175-1

https://www.scopus.com/inward/record.uri?eid=2-s2.0-85216607573&doi=10.1186%2fs12884-025-07175-1&partnerID=40&md5=6f1dc61dfd6cfa647bb74c091bdfa671

DOCUMENT TYPE: Article

PUBLICATION STAGE: Final

OPEN ACCESS: All Open Access; Gold Open Access; Green Open Access

SOURCE: Scopus

Pilkay S., Halevy D., Femia D., Holland S.M., Heiland S., Carroll A.M., Nunes S., Veerman T., Dudak L.

AUTHOR FULL NAMES: Pilkay, Stefanie (56590079700); Halevy, Devorah (58888155200); Femia, Danielle (58888155100); Holland, Sage Malina (59544484100); Heiland, Stephanie (58887595200); Carroll, Andrew M (58971226800); Nunes, Sarah (59099933100); Veerman, Tara (57196279974); Dudak, Leah (57990801000)

56590079700; 58888155200; 58888155100; 59544484100; 58887595200; 58971226800; 59099933100; 57196279974; 57990801000

Adolescent Depression and Cognition Risk for Suicide: An Investigation of Risk Factors and Gene Environment Interactions

(2025) Brain and Behavior, 15 (2), art. no. e70247, Cited 1 times.

DOI: 10.1002/brb3.70247

https://www.scopus.com/inward/record.uri?eid=2-s2.0-85217021026&doi=10.1002%2fbrb3.70247&partnerID=40&md5=3e56181fa237647b79fa56cdec81cf75

DOCUMENT TYPE: Article

PUBLICATION STAGE: Final

OPEN ACCESS: All Open Access; Gold Open Access

SOURCE: Scopus

Carvalho C., Oshri A.

AUTHOR FULL NAMES: Carvalho, Cory (57343253200); Oshri, Assaf (25959713600)

57343253200; 25959713600

The Protective Role of the Autonomic Nervous System in Intergenerational Cycles of Neglect

(2025) Child Psychiatry and Human Development, 56 (2), pp. 556 - 569, Cited 0 times.

DOI: 10.1007/s10578-023-01580-3

https://www.scopus.com/inward/record.uri?eid=2-s2.0-105001065414&doi=10.1007%2fs10578-023-01580-3&partnerID=40&md5=88a10e5ac8796eed5e1eefe188a56790

DOCUMENT TYPE: Article

PUBLICATION STAGE: Final

SOURCE: Scopus

Ji H., Zhu H., Wang Z., Liang H., Chen Y., Liu X., Yuan W., Wu Q., Yuan Z., Miao M.

AUTHOR FULL NAMES: Ji, Honglei (55768222100); Zhu, Haijun (58682348000); Wang, Ziliang (57192100409); Liang, Hong (55742840500); Chen, Yao (57219612023); Liu, Xiao (59727465200); Yuan, Wei (35486224800); Wu, Qihan (7404602342); Yuan, Zhengwei (10641253300); Miao, Maohua (26632021200)

55768222100; 58682348000; 57192100409; 55742840500; 57219612023; 59727465200; 35486224800; 7404602342; 10641253300; 26632021200

Prenatal bisphenol analogs exposure and placental DNA hypomethylation of genes in the PPAR signaling pathway: Insights for bisphenol analogs’ effects on infant anthropometry

(2025) Environmental Research, 266, art. no. 120476, Cited 1 times.

DOI: 10.1016/j.envres.2024.120476

https://www.scopus.com/inward/record.uri?eid=2-s2.0-85210709063&doi=10.1016%2fj.envres.2024.120476&partnerID=40&md5=68e146a6576886bc2e6d13cc574e0614

DOCUMENT TYPE: Article

PUBLICATION STAGE: Final

SOURCE: Scopus

Segura A.-G., Prohens L., Julià L., Amoretti S., RIbero M., Pino-Camacho L., Cano-Escalera G., Mane A., Rodriguez-Jimenez R., Roldan A., Sarró S., Ibañez A., Usall J., Lobo A., Garcia-Rizo C., Cuesta M.J., Parellada M., González-Pinto A., Berrocoso E., Bernardo M., Mas S., Rodríguez N., Perez-Ramos A., Salmeron S., González-Peñas J., Gurriarán X., Farré A., Pousa E., Zorrilla I., Mar-Barrutia L., Trabsa A., Martinez L., Sánchez-Cabezudo Á., Jiménez-López E., Pomarol-Clotet E., Salvador R., Butjosa A., Elena R.-A., Moreno-Izco L., Torres A.M.S., Saiz J., León-Quismondo L., Rivero O., González-Blanco L., De-la-Cámara C.

AUTHOR FULL NAMES: Segura, Alex-González (57209215541); Prohens, Llucia (57246318200); Julià, Laura (57969532300); Amoretti, Silvia (57189219360); RIbero, Maria (59560303600); Pino-Camacho, Laura (59559894700); Cano-Escalera, Guillermo (57222744911); Mane, Anna (16637916100); Rodriguez-Jimenez, Roberto (8724872300); Roldan, Alexandra (56490154800); Sarró, Salvador (6602585245); Ibañez, Angela (7005052960); Usall, Judith (57218668889); Lobo, Antonio (57211735677); Garcia-Rizo, Clemente (24331499300); Cuesta, Manuel Jesus (7102661185); Parellada, Mara (14040702000); González-Pinto, Ana (57209361735); Berrocoso, Esther (6506843024); Bernardo, Miquel (7103259062); Mas, Sergi (6701721228); Rodríguez, Natalia (56072141200); Perez-Ramos, Anaid (59132690700); Salmeron, Sergi (58688882500); González-Peñas, Javier (35336386500); Gurriarán, Xaquín (57194743203); Farré, Adriana (57191034032); Pousa, Esther (7801404365); Zorrilla, Iñaki (48762368900); Mar-Barrutia, Lorea (57074188600); Trabsa, Amira (57314459700); Martinez, Laura (57695898200); Sánchez-Cabezudo, Ángeles (7801333866); Jiménez-López, Estela (57193238919); Pomarol-Clotet, Edith (8873538700); Salvador, Raymond (56219433700); Butjosa, Anna (56230827600); Elena, Rubio-Abadal (59370642600); Moreno-Izco, Lucía (38362049200); Torres, Ana M. Sánchez (56367471000); Saiz, Jeronimo (57225666339); León-Quismondo, Leticia (57221863284); Rivero, Olga (11140735500); González-Blanco, Leticia (56109006700); De-la-Cámara, Concepción (6601907179)

57209215541; 57246318200; 57969532300; 57189219360; 59560303600; 59559894700; 57222744911; 16637916100; 8724872300; 56490154800; 6602585245; 7005052960; 57218668889; 57211735677; 24331499300; 7102661185; 14040702000; 57209361735; 6506843024; 7103259062; 6701721228; 56072141200; 59132690700; 58688882500; 35336386500; 57194743203; 57191034032; 7801404365; 48762368900; 57074188600; 57314459700; 57695898200; 7801333866; 57193238919; 8873538700; 56219433700; 56230827600; 59370642600; 38362049200; 56367471000; 57225666339; 57221863284; 11140735500; 56109006700; 6601907179

Methylation profile scores of environmental exposures and risk of relapse after a first episode of schizophrenia

(2025) European Neuropsychopharmacology, 94, pp. 4 - 15, Cited 1 times.

DOI: 10.1016/j.euroneuro.2025.02.003

https://www.scopus.com/inward/record.uri?eid=2-s2.0-85217964975&doi=10.1016%2fj.euroneuro.2025.02.003&partnerID=40&md5=7de2f0db9966306db646d2987029be3c

DOCUMENT TYPE: Article

PUBLICATION STAGE: Final

SOURCE: Scopus

León I., Góngora D., Rodrigo M.J., Herrero-Roldán S., López Rodríguez M., Mitchell C., Fisher J., Iturria-Medina Y.

AUTHOR FULL NAMES: León, Inmaculada (7003302900); Góngora, Daylín (56874151900); Rodrigo, María José (55710119600); Herrero-Roldán, Silvia (57304447200); López Rodríguez, Maykel (57194697698); Mitchell, Colter (36198825600); Fisher, Jonah (57221604294); Iturria-Medina, Yasser (16506904600)

7003302900; 56874151900; 55710119600; 57304447200; 57194697698; 36198825600; 57221604294; 16506904600

Maternal epigenetic index links early neglect to later neglectful care and other psychopathological, cognitive, and bonding effects

(2025) Clinical Epigenetics, 17 (1), art. no. 46, Cited 0 times.

DOI: 10.1186/s13148-025-01839-7

https://www.scopus.com/inward/record.uri?eid=2-s2.0-86000724423&doi=10.1186%2fs13148-025-01839-7&partnerID=40&md5=4ee72ae121f693783c2884b7e2e7fe67

DOCUMENT TYPE: Article

PUBLICATION STAGE: Final

OPEN ACCESS: All Open Access; Gold Open Access

SOURCE: Scopus

Donno V., Prats P., Rodriguez I., Polyzos N.P.

AUTHOR FULL NAMES: Donno, Valeria (57218382241); Prats, Pilar (19035857100); Rodriguez, Ignacio (7102747510); Polyzos, Nikolaos P. (11839749200)

57218382241; 19035857100; 7102747510; 11839749200

First-trimester uterine artery pulsatility index and preeclampsia risk in pregnancies after artificial frozen embryo transfer: analysis of over 27,000 pregnancies

(2025) American Journal of Obstetrics and Gynecology, 232 (5), pp. 464.e1 - 464.e9, Cited 1 times.

DOI: 10.1016/j.ajog.2024.10.033

https://www.scopus.com/inward/record.uri?eid=2-s2.0-85211352373&doi=10.1016%2fj.ajog.2024.10.033&partnerID=40&md5=2fa40b02df452bf92d6a5b347e999053

DOCUMENT TYPE: Article

PUBLICATION STAGE: Final

SOURCE: Scopus

Wang K., Zhang L., Li Q., Xu S., Wang P., Shi H., Zhang Y., Li J.

AUTHOR FULL NAMES: Wang, Kuancheng (59360962600); Zhang, Liyi (57284114600); Li, Qiang (57191756782); Xu, Siqi (59360785800); Wang, Pengpeng (57208321715); Shi, Huijing (55448174400); Zhang, Yunhui (56716591300); Li, Jiufeng (57190425350)

59360962600; 57284114600; 57191756782; 59360785800; 57208321715; 55448174400; 56716591300; 57190425350

The effect of PM2.5 exposure on placenta and its associated metabolites: A birth cohort study

(2025) Ecotoxicology and Environmental Safety, 292, art. no. 117891, Cited 1 times.

DOI: 10.1016/j.ecoenv.2025.117891

https://www.scopus.com/inward/record.uri?eid=2-s2.0-85217954802&doi=10.1016%2fj.ecoenv.2025.117891&partnerID=40&md5=9a1be8398c5a52c09ed2f63c83e9bc6f

DOCUMENT TYPE: Article

PUBLICATION STAGE: Final

OPEN ACCESS: All Open Access; Gold Open Access

SOURCE: Scopus

Lax E.

AUTHOR FULL NAMES: Lax, Elad (26028014100)

26028014100

Short-term maternal oxytocin administration may protect against poor outcomes of adverse childhood experiences in the offspring

(2025) Neurotherapeutics, art. no. e00605, Cited 0 times.

DOI: 10.1016/j.neurot.2025.e00605

https://www.scopus.com/inward/record.uri?eid=2-s2.0-105004363706&doi=10.1016%2fj.neurot.2025.e00605&partnerID=40&md5=793dc2761061af5f4a5255f9b70ada10

DOCUMENT TYPE: Article

PUBLICATION STAGE: Article in press

OPEN ACCESS: All Open Access; Gold Open Access

SOURCE: Scopus

ProQuest Dissertations and Theses Global

Use of Epigenetic Biomarkers to Study the Impacts of Prenatal Endocrine-Disrupting Chemical Exposure in Childhood

Author: Khodasevich, Dennis

Publication info: Khodasevich, Dennis.

https://ezproxy.lib.ucalgary.ca/login?qurl=https%3A%2F%2Fwww.proquest.com%2Fdissertations-theses%2Fuse-epigenetic-biomarkers-study-impacts-prenatal%2Fdocview%2F3111078617%2Fse-2%3Faccountid%3D9838

Links: https://ucalgary.primo.exlibrisgroup.com/openurl/01UCALG_INST/01UCALG_INST:UCALGARY?genre=dissertations&amp;atitle=&amp;author=Khodasevich%2C+Dennis&amp;volume=&amp;issue=&amp;spage=&amp;date=2024&amp;rft.btitle=&amp;rft.title=Use+of+Epigenetic+Biomarkers+to+Study+the+Impacts+of+Prenatal+Endocrine-Disrupting+Chemical+Exposure+in+Childhood&amp;rft.jtitle=&amp;issn=&amp;isbn=9798384452867&amp;sid=ProQuest+Dissertations+%26+Theses+Global_

____________________________________________________________

A Journey to Healing: Identifying Intergenerational Trauma, ACEs, Racial Trauma and PTSD in Mothers of Color

Author: Greene, Nadine R.

Publication info: Greene, Nadine R.

https://ezproxy.lib.ucalgary.ca/login?qurl=https%3A%2F%2Fwww.proquest.com%2Fdissertations-theses%2Fjourney-healing-identifying-intergenerational%2Fdocview%2F2904459352%2Fse-2%3Faccountid%3D9838

Links: https://ucalgary.primo.exlibrisgroup.com/openurl/01UCALG_INST/01UCALG_INST:UCALGARY?genre=dissertations&amp;atitle=&amp;author=Greene%2C+Nadine+R.&amp;volume=&amp;issue=&amp;spage=&amp;date=2023&amp;rft.btitle=&amp;rft.title=A+Journey+to+Healing%3A+Identifying+Intergenerational+Trauma%2C+ACEs%2C+Racial+Trauma+and+PTSD+in+Mothers+of+Color&amp;rft.jtitle=&amp;issn=&amp;isbn=9798381181968&amp;sid=ProQuest+Dissertations+%26+Theses+Global_

____________________________________________________________

Experiences of Childhood Adversity and Racial Discrimination Among Black Young Adults

Author: Rowell, Tiffany A.

Publication info: Rowell, Tiffany A.

https://ezproxy.lib.ucalgary.ca/login?qurl=https%3A%2F%2Fwww.proquest.com%2Fdissertations-theses%2Fexperiences-childhood-adversity-racial%2Fdocview%2F2858089500%2Fse-2%3Faccountid%3D9838

Links: https://ucalgary.primo.exlibrisgroup.com/openurl/01UCALG_INST/01UCALG_INST:UCALGARY?genre=dissertations&amp;atitle=&amp;author=Rowell%2C+Tiffany+A.&amp;volume=&amp;issue=&amp;spage=&amp;date=2023&amp;rft.btitle=&amp;rft.title=Experiences+of+Childhood+Adversity+and+Racial+Discrimination+Among+Black+Young+Adults&amp;rft.jtitle=&amp;issn=&amp;isbn=9798380102339&amp;sid=ProQuest+Dissertations+%26+Theses+Global_

____________________________________________________________

Life-Course Pathways to Carcinogenesis for Persistent Endocrine-Disrupting Chemicals

Author: Kaufman, John A.

Publication info: Kaufman, John A.

https://ezproxy.lib.ucalgary.ca/login?qurl=https%3A%2F%2Fwww.proquest.com%2Fdissertations-theses%2Flife-course-pathways-carcinogenesis-persistent%2Fdocview%2F2828281182%2Fse-2%3Faccountid%3D9838

Links: https://ucalgary.primo.exlibrisgroup.com/openurl/01UCALG_INST/01UCALG_INST:UCALGARY?genre=dissertations&amp;atitle=&amp;author=Kaufman%2C+John+A.&amp;volume=&amp;issue=&amp;spage=&amp;date=2023&amp;rft.btitle=&amp;rft.title=Life-Course+Pathways+to+Carcinogenesis+for+Persistent+Endocrine-Disrupting+Chemicals&amp;rft.jtitle=&amp;issn=&amp;isbn=9798379717766&amp;sid=ProQuest+Dissertations+%26+Theses+Global_

____________________________________________________________

Risk and Resilience in the Intergenerational Impacts of Childhood Trauma Among Black Americans: Moderating Roles of Epigenetic Aging and the Gut Microbiome

Author: McKenna, Brooke G.

Publication info: McKenna, Brooke G.

https://ezproxy.lib.ucalgary.ca/login?qurl=https%3A%2F%2Fwww.proquest.com%2Fdissertations-theses%2Frisk-resilience-intergenerational-impacts%2Fdocview%2F2828163958%2Fse-2%3Faccountid%3D9838

Links: https://ucalgary.primo.exlibrisgroup.com/openurl/01UCALG_INST/01UCALG_INST:UCALGARY?genre=dissertations&amp;atitle=&amp;author=McKenna%2C+Brooke+G.&amp;volume=&amp;issue=&amp;spage=&amp;date=2023&amp;rft.btitle=&amp;rft.title=Risk+and+Resilience+in+the+Intergenerational+Impacts+of+Childhood+Trauma+Among+Black+Americans%3A+Moderating+Roles+of+Epigenetic+Aging+and+the+Gut+Microbiome&amp;rft.jtitle=&amp;issn=&amp;isbn=9798379716417&amp;sid=ProQuest+Dissertations+%26+Theses+Global_

____________________________________________________________

Telomere Length: A Biomarker of Aging in Neurodevelopment and Neuropyschological Performance for Minoritized Populations

Author: McLester-Davis, Lauren Wendelle Yowelunh

Publication info: McLester-Davis, Lauren Wendelle Yowelunh.

https://ezproxy.lib.ucalgary.ca/login?qurl=https%3A%2F%2Fwww.proquest.com%2Fdissertations-theses%2Ftelomere-length-biomarker-aging-neurodevelopment%2Fdocview%2F2821626703%2Fse-2%3Faccountid%3D9838

Links: https://ucalgary.primo.exlibrisgroup.com/openurl/01UCALG_INST/01UCALG_INST:UCALGARY?genre=dissertations&amp;atitle=&amp;author=McLester-Davis%2C+Lauren+Wendelle+Yowelunh&amp;volume=&amp;issue=&amp;spage=&amp;date=2023&amp;rft.btitle=&amp;rft.title=Telomere+Length%3A+A+Biomarker+of+Aging+in+Neurodevelopment+and+Neuropyschological+Performance+for+Minoritized+Populations&amp;rft.jtitle=&amp;issn=&amp;isbn=9798379611026&amp;sid=ProQuest+Dissertations+%26+Theses+Global_

____________________________________________________________

The Structural and Social Determinants of Intergenerational Health Inequities: How State Policy Contexts and Discrimination Shape Birth Outcomes

Author: Stolte, Allison

Publication info: Stolte, Allison.

https://ezproxy.lib.ucalgary.ca/login?qurl=https%3A%2F%2Fwww.proquest.com%2Fdissertations-theses%2Fstructural-social-determinants-intergenerational%2Fdocview%2F2819213876%2Fse-2%3Faccountid%3D9838

Links: https://ucalgary.primo.exlibrisgroup.com/openurl/01UCALG_INST/01UCALG_INST:UCALGARY?genre=dissertations&amp;atitle=&amp;author=Stolte%2C+Allison&amp;volume=&amp;issue=&amp;spage=&amp;date=2023&amp;rft.btitle=&amp;rft.title=The+Structural+and+Social+Determinants+of+Intergenerational+Health+Inequities%3A+How+State+Policy+Contexts+and+Discrimination+Shape+Birth+Outcomes&amp;rft.jtitle=&amp;issn=&amp;isbn=9798379572525&amp;sid=ProQuest+Dissertations+%26+Theses+Global_

____________________________________________________________

Gestational Epigenetic Aging: Prenatal Influences and Early Life Associations

Author: Pawlowski, Emilia

Publication info: Pawlowski, Emilia.

https://ezproxy.lib.ucalgary.ca/login?qurl=https%3A%2F%2Fwww.proquest.com%2Fdissertations-theses%2Fgestational-epigenetic-aging-prenatal-influences%2Fdocview%2F2808487386%2Fse-2%3Faccountid%3D9838

Links: https://ucalgary.primo.exlibrisgroup.com/openurl/01UCALG_INST/01UCALG_INST:UCALGARY?genre=dissertations&amp;atitle=&amp;author=Pawlowski%2C+Emilia&amp;volume=&amp;issue=&amp;spage=&amp;date=2023&amp;rft.btitle=&amp;rft.title=Gestational+Epigenetic+Aging%3A+Prenatal+Influences+and+Early+Life+Associations&amp;rft.jtitle=&amp;issn=&amp;isbn=9798379454173&amp;sid=ProQuest+Dissertations+%26+Theses+Global_

____________________________________________________________

A Biocultural Approach to Understanding the Impacts of Psychosocial Stress

Author: Quinn, Edward B.

Publication info: Quinn, Edward B.

https://ezproxy.lib.ucalgary.ca/login?qurl=https%3A%2F%2Fwww.proquest.com%2Fdissertations-theses%2Fbiocultural-approach-understanding-impacts%2Fdocview%2F2932843783%2Fse-2%3Faccountid%3D9838

Links: https://ucalgary.primo.exlibrisgroup.com/openurl/01UCALG_INST/01UCALG_INST:UCALGARY?genre=dissertations&amp;atitle=&amp;author=Quinn%2C+Edward+B.&amp;volume=&amp;issue=&amp;spage=&amp;date=2022&amp;rft.btitle=&amp;rft.title=A+Biocultural+Approach+to+Understanding+the+Impacts+of+Psychosocial+Stress&amp;rft.jtitle=&amp;issn=&amp;isbn=9798381730609&amp;sid=ProQuest+Dissertations+%26+Theses+Global_

____________________________________________________________

Three Stories About Intergenerational Trauma

Author: Rivera, Luisa Maria

Publication info: Rivera, Luisa Maria.

https://ezproxy.lib.ucalgary.ca/login?qurl=https%3A%2F%2Fwww.proquest.com%2Fdissertations-theses%2Fthree-stories-about-intergenerational-trauma%2Fdocview%2F2784393910%2Fse-2%3Faccountid%3D9838

Links: https://ucalgary.primo.exlibrisgroup.com/openurl/01UCALG_INST/01UCALG_INST:UCALGARY?genre=dissertations&amp;atitle=&amp;author=Rivera%2C+Luisa+Maria&amp;volume=&amp;issue=&amp;spage=&amp;date=2022&amp;rft.btitle=&amp;rft.title=Three+Stories+About+Intergenerational+Trauma&amp;rft.jtitle=&amp;issn=&amp;isbn=9798371982063&amp;sid=ProQuest+Dissertations+%26+Theses+Global_

____________________________________________________________

Evaluating Stochastic Epigenetic Mutations as Potential Mechanism Translating Psychosocial Stressors into Risk for Postpartum Depression

Author: Todd, Julianne E.

Publication info: Todd, Julianne E.

https://ezproxy.lib.ucalgary.ca/login?qurl=https%3A%2F%2Fwww.proquest.com%2Fdissertations-theses%2Fevaluating-stochastic-epigenetic-mutations-as%2Fdocview%2F2784391194%2Fse-2%3Faccountid%3D9838

Links: https://ucalgary.primo.exlibrisgroup.com/openurl/01UCALG_INST/01UCALG_INST:UCALGARY?genre=dissertations&amp;atitle=&amp;author=Todd%2C+Julianne+E.&amp;volume=&amp;issue=&amp;spage=&amp;date=2022&amp;rft.btitle=&amp;rft.title=Evaluating+Stochastic+Epigenetic+Mutations+as+Potential+Mechanism+Translating+Psychosocial+Stressors+into+Risk+for+Postpartum+Depression&amp;rft.jtitle=&amp;issn=&amp;isbn=9798371980991&amp;sid=ProQuest+Dissertations+%26+Theses+Global_

____________________________________________________________

Intergenerational Associations between Parental Adversity and Offspring Health Outcomes in African-American Families

Author: Sweeting, Josiah A.

Publication info: Sweeting, Josiah A.

https://ezproxy.lib.ucalgary.ca/login?qurl=https%3A%2F%2Fwww.proquest.com%2Fdissertations-theses%2Fintergenerational-associations-between-parental%2Fdocview%2F2727650404%2Fse-2%3Faccountid%3D9838

Links: https://ucalgary.primo.exlibrisgroup.com/openurl/01UCALG_INST/01UCALG_INST:UCALGARY?genre=dissertations&amp;atitle=&amp;author=Sweeting%2C+Josiah+A.&amp;volume=&amp;issue=&amp;spage=&amp;date=2022&amp;rft.btitle=&amp;rft.title=Intergenerational+Associations+between+Parental+Adversity+and+Offspring+Health+Outcomes+in+African-American+Families&amp;rft.jtitle=&amp;issn=&amp;isbn=9798352937327&amp;sid=ProQuest+Dissertations+%26+Theses+Global_

____________________________________________________________

A Dimensional Perspective of Adverse Childhood Experiences: Relations to Adult Health and Neurocognitive Characteristics in Veterans with Comorbid PTSD and Alcohol Use Disorder

Author: Padua, Mayra A. Gomez

Publication info: Padua, Mayra A. Gomez.

https://ezproxy.lib.ucalgary.ca/login?qurl=https%3A%2F%2Fwww.proquest.com%2Fdissertations-theses%2Fdimensional-perspective-adverse-childhood%2Fdocview%2F2594707385%2Fse-2%3Faccountid%3D9838

Links: https://ucalgary.primo.exlibrisgroup.com/openurl/01UCALG_INST/01UCALG_INST:UCALGARY?genre=dissertations&amp;atitle=&amp;author=Padua%2C+Mayra+A.+Gomez&amp;volume=&amp;issue=&amp;spage=&amp;date=2021&amp;rft.btitle=&amp;rft.title=A+Dimensional+Perspective+of+Adverse+Childhood+Experiences%3A+Relations+to+Adult+Health+and+Neurocognitive+Characteristics+in+Veterans+with+Comorbid+PTSD+and+Alcohol+Use+Disorder&amp;rft.jtitle=&amp;issn=&amp;isbn=9798492728649&amp;sid=ProQuest+Dissertations+%26+Theses+Global_

____________________________________________________________

Institutional Racism and Maternal and Child Health: Exploring the Relationship Between Correctional Control, Maternal Morbidity, and Preterm Birth

Author: Ramsey, Lauren Meta

Publication info: Ramsey, Lauren Meta.

https://ezproxy.lib.ucalgary.ca/login?qurl=https%3A%2F%2Fwww.proquest.com%2Fdissertations-theses%2Finstitutional-racism-maternal-child-health%2Fdocview%2F2584301760%2Fse-2%3Faccountid%3D9838

Links: https://ucalgary.primo.exlibrisgroup.com/openurl/01UCALG_INST/01UCALG_INST:UCALGARY?genre=dissertations&amp;atitle=&amp;author=Ramsey%2C+Lauren+Meta&amp;volume=&amp;issue=&amp;spage=&amp;date=2021&amp;rft.btitle=&amp;rft.title=Institutional+Racism+and+Maternal+and+Child+Health%3A+Exploring+the+Relationship+Between+Correctional+Control%2C+Maternal+Morbidity%2C+and+Preterm+Birth&amp;rft.jtitle=&amp;issn=&amp;isbn=9798460452903&amp;sid=ProQuest+Dissertations+%26+Theses+Global_

____________________________________________________________

Subclinical Stress on Obesity Among Obese African American Women

Author: Idehen, Dr Nosakhare O.

Publication info: Idehen, Dr Nosakhare O.

https://ezproxy.lib.ucalgary.ca/login?qurl=https%3A%2F%2Fwww.proquest.com%2Fdissertations-theses%2Fsubclinical-stress-on-obesity-among-obese-african%2Fdocview%2F2508666304%2Fse-2%3Faccountid%3D9838

Links: https://ucalgary.primo.exlibrisgroup.com/openurl/01UCALG_INST/01UCALG_INST:UCALGARY?genre=dissertations&amp;atitle=&amp;author=Idehen%2C+Dr+Nosakhare+O.&amp;volume=&amp;issue=&amp;spage=&amp;date=2021&amp;rft.btitle=&amp;rft.title=Subclinical+Stress+on+Obesity+Among+Obese+African+American+Women&amp;rft.jtitle=&amp;issn=&amp;isbn=9798597009186&amp;sid=ProQuest+Dissertations+%26+Theses+Global_

____________________________________________________________

Quality of Attention of the Mother in Infancy and Early Childhood Sets the Stage for Development throughout the Child’s Life

Author: Suettmann, Margot

Publication info: Suettmann, Margot.

https://ezproxy.lib.ucalgary.ca/login?qurl=https%3A%2F%2Fwww.proquest.com%2Fdissertations-theses%2Fquality-attention-mother-infancy-early-childhood%2Fdocview%2F2418591198%2Fse-2%3Faccountid%3D9838

Links: https://ucalgary.primo.exlibrisgroup.com/openurl/01UCALG_INST/01UCALG_INST:UCALGARY?genre=dissertations&amp;atitle=&amp;author=Suettmann%2C+Margot&amp;volume=&amp;issue=&amp;spage=&amp;date=2020&amp;rft.btitle=&amp;rft.title=Quality+of+Attention+of+the+Mother+in+Infancy+and+Early+Childhood+Sets+the+Stage+for+Development+throughout+the+Child%E2%80%99s+Life&amp;rft.jtitle=&amp;issn=&amp;isbn=9798641272931&amp;sid=ProQuest+Dissertations+%26+Theses+Global_

____________________________________________________________

Examining the Impact of Pregnant Black Women's Adverse Childhood Experiences Through Maternal Health and Birth Outcomes

Author: Rowell, Tiffany A.

Publication info: Rowell, Tiffany A.

https://ezproxy.lib.ucalgary.ca/login?qurl=https%3A%2F%2Fwww.proquest.com%2Fdissertations-theses%2Fexamining-impact-pregnant-black-womens-adverse%2Fdocview%2F2405307126%2Fse-2%3Faccountid%3D9838

Links: https://ucalgary.primo.exlibrisgroup.com/openurl/01UCALG_INST/01UCALG_INST:UCALGARY?genre=dissertations&amp;atitle=&amp;author=Rowell%2C+Tiffany+A.&amp;volume=&amp;issue=&amp;spage=&amp;date=2020&amp;rft.btitle=&amp;rft.title=Examining+the+Impact+of+Pregnant+Black+Women%27s+Adverse+Childhood+Experiences+Through+Maternal+Health+and+Birth+Outcomes&amp;rft.jtitle=&amp;issn=&amp;isbn=9798643176855&amp;sid=ProQuest+Dissertations+%26+Theses+Global_

____________________________________________________________

The Transgenerational Impact of Maternal Early Life Adversity on Infant Development

Author: Jones, Christopher William

Publication info: Jones, Christopher William.

https://ezproxy.lib.ucalgary.ca/login?qurl=https%3A%2F%2Fwww.proquest.com%2Fdissertations-theses%2Ftransgenerational-impact-maternal-early-life%2Fdocview%2F2313355473%2Fse-2%3Faccountid%3D9838

Links: https://ucalgary.primo.exlibrisgroup.com/openurl/01UCALG_INST/01UCALG_INST:UCALGARY?genre=dissertations&amp;atitle=&amp;author=Jones%2C+Christopher+William&amp;volume=&amp;issue=&amp;spage=&amp;date=2019&amp;rft.btitle=&amp;rft.title=The+Transgenerational+Impact+of+Maternal+Early+Life+Adversity+on+Infant+Development&amp;rft.jtitle=&amp;issn=&amp;isbn=9781088386019&amp;sid=ProQuest+Dissertations+%26+Theses+Global_

____________________________________________________________

Contact us at: http://about.proquest.com/go/pqissupportcontact

Database copyright © 2025 ProQuest LLC. All rights reserved.

Terms and Conditions: https://www.proquest.com/info/termsAndConditions
